# Adjunctive Local Agents to Subgingival Instrumentation in the Treatment of Periodontitis: A Review of Recent Clinical Trials and Future Perspectives

**DOI:** 10.3390/pharmaceutics17060697

**Published:** 2025-05-26

**Authors:** William G. Boivin, Maxwell T. Cory, Ioannis Kormas, Larry F. Wolff

**Affiliations:** Division of Periodontology, Department of Developmental and Surgical Sciences, School of Dentistry, University of Minnesota, Minneapolis, MN 55455, USA

**Keywords:** local delivery, antimicrobials, photodynamic therapy, periodontitis

## Abstract

The purpose of this narrative review is to identify and present clinical trials published in the last five years on local delivery agents used as adjuncts to subgingival instrumentation in the non-surgical management of periodontitis. Epidemiological studies have shown that periodontitis is highly prevalent in the general population. Treatment is usually based on mechanical removal of contaminants from the root surface followed by long-term supportive care, resulting in decreased occurrence of tooth loss. Clinical health is not always achieved at all sites, leading to research efforts by researchers to find adjunctive agents to help improve the periodontal condition. This review aims to present the most recent developments in local adjunctive agents for the non-surgical treatment of periodontitis. Therapies used included antimicrobial photodynamic therapy as well as antimicrobial and biomodulating compounds. A search in PubMed was conducted to identify the most recent randomized controlled trials relating to locally delivered adjunctive agents in periodontitis treatment beyond traditional therapies such as chlorhexidine, minocycline and doxycycline. Thirty-one articles published in the last five years were included. The most current evidence from human trials supports that, despite the high variability in experimental protocols, there may be a clinical benefit to antimicrobial photodynamic therapy and gels carrying sodium hypochlorite, melatonin, tea tree oil and *Aloe vera*. Most recently, advances in nanotechnology, including liposomes, present an avenue forward to potentially increase the effectiveness of current and future local delivery agents in the non-surgical treatment of periodontitis.

## 1. Introduction

Periodontitis is a highly prevalent disease, with an estimated 46% of the United States population being affected [1], with the latest estimated worldwide prevalence of severe periodontitis reaching 12.5% [2]. The disease is defined by chronic inflammation that in advanced stages leads to damage and destruction of the periodontal ligament and surrounding alveolar bone, thus weakening tooth support [3]. If allowed to progress without intervention, periodontitis can lead to loss of teeth, a significant decrease in masticatory function and worsen overall quality of life [4]. Non-surgical therapy followed by a strict maintenance protocol presents the least invasive option to treat the disease, but there are limitations to what can be achieved with this approach [5]. Initial subgingival instrumentation (SI) in non-surgical therapy around teeth affected by periodontitis will yield a reduction in probing periodontal pocket depths (PPDs) of 1 mm on average, while the most severe sites presenting PPDs of 7 mm or more may present an improvement of 2 mm [6]. This may result in persistent periodontal pockets and an increased risk of tooth loss [7]. These residual pockets therefore may be managed with repeated SI or more invasive treatment modalities such as periodontal surgery [5]. According to the publication resulting from the 2017 American Academy of Periodontology World Workshop, the periodontal condition is considered stable if no PPDs deeper than 4 mm are found, and if no 4 mm pocket presents clinical signs of inflammation, such as bleeding on probing (BOP) [8]. Considerable research efforts have been directed toward achieving a greater PPD reduction and decrease in BOP following non-surgical SI. For instance, adjunctive locally delivered agents, which are deposited directly into periodontal pockets, have been used to help achieve the desired level of disease resolution in periodontitis sites [9]. Compared to systemic antibiotic treatment, targeted local delivery reduces the risk of adverse events associated with systemic antibiotic administration and an increased concentration of antibiotic at the site of the disease [10]. Local delivery agents that have been investigated thus far are mostly antimicrobial, such as minocycline microspheres, doxycycline and chlorhexidine (CHX) [11,12,13]. A meta-analysis of locally delivered agents found that a statistically significant increase in clinical attachment level (CAL) and reduction in PPDs may be achieved when used as an adjunct to SI, but this difference was less than 1 mm on average [9]. Evidence supports that clinicians cannot accurately detect a difference of less than 1 mm on clinical examination of human patients using a standard periodontal probe [14]. New evidence is emerging in the field of locally delivered compounds and their accompanied vehicles, including compounds at the center of antimicrobial photodynamic therapy (aPDT) therapy. The purpose of this narrative review is to identify and present clinical trials published in the last five years on local delivery agents used as adjuncts to SI in the non-surgical management of periodontitis.

## 2. Material and Methods

A search of the PubMed database was conducted using the following terms and Boolean operators:

((“periodontitis”[All Fields] OR “periodontal disease”[All Fields] OR “scaling and root planing”[All Fields]) AND (“local delivery”[All Fields] OR “topical delivery”[All Fields] OR “gel”[All Fields] OR “adjunctive agent”[All Fields] OR “antimicrobial”[All Fields])) AND ((y_5[Filter]) AND (randomized controlled trial[Filter]) AND (fft[Filter])).

**Figure 1:**
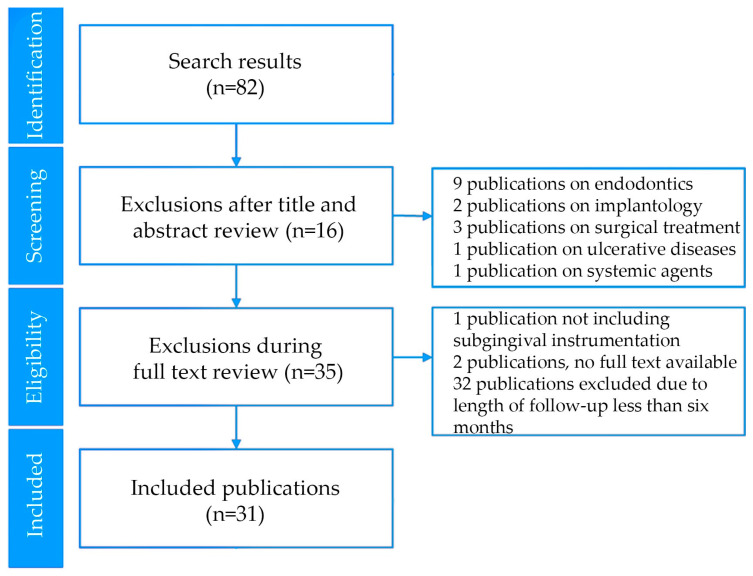
Literature search results, reviewing process and reasons for exclusions. The results from the search on 12 January 2025 included only randomized controlled trials in humans published in the last five years in the English language. Initial screening allowed the inclusion of trials on the treatment of periodontitis. Eligibility was then evaluated by acquiring full texts to identify and exclude publications that did not include non-surgical instrumentation or did not follow patients for at least six months after treatment.

Literature search results, reviewing process and reasons for exclusions are presented in Figure 1. The search was limited to randomized controlled trials published in the last five years with a full text available. The search on 12 January 2025 identified 82 potential publications. Sixteen publications were excluded in the screening phase because they did not include non-surgical treatment of periodontitis using locally delivered agents. Of the remaining articles, two were not available in full text. Thirty-two trials with a duration of less than six months were also excluded as part of the full text review, leaving 31 articles to be included in this narrative review.

## 3. Results

### 3.1. Adjunctive Antimicrobial Photodynamic Therapy

The thirteen articles on adjunctive antimicrobial photodynamic therapy published in the last five years included a variety of photosensitizing agents and diode lasers. Protocols varied between repeated and single application approaches. This modality was also investigated in the treatment of residual pockets in patients enrolled in periodontal maintenance therapy and patients in their initial phase of treatment.

The methods and results of clinical trials on adjunctive antimicrobial photodynamic therapy with SI are presented in Table 1. Two studies found that during initial treatment of periodontitis with SI, repeated aPDT presented greater improvements in clinical parameters than the control groups [15,16] while two studies found no advantage when measuring clinical parameters for the aPDT group [17,18]. Al-Khureif et al. found that repeated use of phenothiazine chloride aPDT led to a greater improvement in CAL (*p* < 0.001) and PPD (*p* = 0.0013) after six months of follow-up compared to patients randomized to be treated with systemic antibiotic adjuncts and SI in the control group [15]. Sukumar et al. compared SI with adjunctive indocyanine green (ICG) aPDT to SI alone and found that BOP (*p* < 0.05), PPD (*p* < 0.001) and CAL (*p* < 0.05) improvements were greater in the test group compared to the control [16]. Cláudio et al. found no statistically significant differences between the aPDT group and the control regarding PPD reduction, CAL, BOP and the number of sites with deep pockets (*p* > 0.05) after six months of follow-up [17]. On the other hand, Annunziata et al. observed that randomized sites treated twice with adjunctive ICG aPDT did as well as control sites having received adjunctive ICG irrigation with SI (*p* > 0.05) [18]. No advantage was identified in the test group when measuring PPD, CAL and BOP six months after SI (*p* > 0.05). Two studies compared repeated application of adjunctive aPDT in residual pockets seen in patients enrolled in supportive periodontal therapy [19,20]. Andere et al. compared repeated SI with adjunctive aPDT using methylene blue to open flap debridement (OFD) in the control group [19]. The surgical modality provided a greater PPD reduction at initially deep sites (*p* = 0.001), with also a greater risk for gingival recession (*p* = 0.001) and dentin hypersensitivity (*p* = 0.03) compared to the test group. Costa and coworkers compared adjunctive aPDT using ICG to a placebo control group using a sham aPDT application with saline irrigation in addition to SI [20]. The test group was found to show a greater reduction in BOP (*p* = 0.046), as well as a reduction in the periodontal inflamed surface area index (*p* = 0.001). Microbiological parameters were also measured using quantitative polymerase chain reaction, showing a greater reduction in bacterial levels of *Porphyromonas gingivalis* (*p* = 0.006) and *Aggregatibacter actinomycetemcomitans* (*p* = 0.015) in the test group.

Two reports presented results following the single application of adjunctive aPDT to initial SI in otherwise healthy periodontitis patients [21,22]. Niazi et al. included a test group using a *Salvadora persica* gel as an adjunctive local delivery group, while another test group received ICG-based aPDT [21]. The adjunctive aPDT group showed a greater PPD reduction and CAL gain (*p* < 0.05) at six months compared to SI alone and to the locally delivered gel group in initially deep pockets. The local delivery group presented a greater reduction in BOP (*p* < 0.05) within the same follow-up time compared to both aPDT and SI alone. Katsikanis et al. compared adjunctive aPDT using methylene blue to the SI alone (control) as well as an adjunctive diode laser group [22]. Results showed that PD, BOP and CAL improvements were similar between all three protocols at the six-month clinical examination (*p* > 0.05).

Diabetic patients were selected for two trials conducted during the initial treatment of periodontitis [23,24]. Al-Momani separated patients into three groups according to their glycated hemoglobin percentage (A1c) values seen on an initial blood test [23]. The first group included patients who did not have diabetes, the second group included type 2 diabetic patients with what was considered by the authors as a reasonably well controlled glycemia (A1c between 6 and 10%) and a third group consisted of patients with uncontrolled diabetes (A1c greater than 10%). Within each group, a split mouth design was used to compare adjunctive aPDT to SI alone. They found that aPDT led to a greater CAL gain and PPD reduction after six months (*p* < 0.05) in the otherwise healthy patients. Poorly controlled diabetics showed greater improvement in CAL and BOP following the aPDT protocol compared to the control (*p* < 0.05). In the 6–10% A1c group, all three parameters showed greater improvements at the six-month examination (*p* < 0.05). When investigating type 1 diabetics undergoing initial periodontal treatment compared to healthy controls, Cunha and coworkers found that CAL, BOP and PPD improved more in normoglycemic patients compared to the type 1 diabetics (*p* < 0.05) [24]. These investigators used repeated adjunctive application of methylene blue aPDT. Within the same health status, both treatment and control performed similarly (*p* > 0.05).

In the population of type 2 diabetic patients recruited by Cláudio et al., the adjunctive aPDT group did as well as the group receiving SI alone regarding improvements in PPD, CAL and BOP (*p* > 0.05) [25]. De Araújo et al. found that the gingival bleeding index was the only clinical parameter presenting a statistically significant improvement (*p* = 0.041) in favor of the test group undergoing chloro aluminum phthalocyanine aPDT treatment [26]. Schär tested adjunctive phenothiazine chloride–based aPDT in otherwise healthy periodontitis patients and found a statistically greater reduction in BOP (*p* < 0.05) in the test group compared to the SI alone group in the treatment of residual pockets found during supportive periodontal therapy [27].

### 3.2. Adjunctive Locally Delivered Agents

Eighteen publications were included regarding targeted periodontal therapy applied at periodontitis sites. These agents included antibiotics, immune modulating and bone metabolism modifying medications. They also included protocols evaluating amino acid gels and antimicrobials like sodium hypochlorite (NaOCl).

Methods and results of clinical trials on adjunctive locally delivered agents with SI are displayed in Table 2. Barahim et al. investigated an adjunctive ozone gel in type 2 diabetics and found that the application of the gel through a specially made stent performed as well as SI alone except for the postoperative pain scored on a visual analog scale (VAS) seven days after the procedure (*p* = 0.017) [28]. They also found a significantly improved periodontal ligament width (reduced) seen on standardized periapical radiographs (*p* = 0.014) after six months of follow-up in the test group.

A report by Gonde et al. showed that intrabony defects treated with SI and 1% melatonin gel had greater bone fill and bone volume seen on cone beam computed tomography (CBCT) at six months of follow-up (*p* < 0.05) [29]. They also found PPD and CAL showed a statistically greater improvement in the adjunctive melatonin group compared to SI alone (*p* < 0.05). Iorio-Siciliano et al. evaluated the combination of minimally invasive nonsurgical therapy (MINST) with a 0.95% NaOCl gel containing an amino acid buffer. The test group had a statistically significant decreased chance of residual PPD of 5 mm or greater with BOP compared to MINST alone (*p* = 0.001) [30].

Qamar et al. tested an uncalibrated adjunctive *Aloe vera* gel to SI, while another test group received aPDT using ICG [31]. The adjunctive gel performed significantly better than adjunctive aPDT and SI alone while measuring improvements in BOP, CAL and PPD (*p* < 0.05). Both the aPDT group and the *Aloe vera* gel group presented reductions in pro-inflammatory cytokines (interleukin-6, interleukin-8 and tumor necrosis factor alpha) lasting up to six months (*p* < 0.05).

Local delivery of bisphosphonates was investigated by Raj et al. [32]. Most clinical and radiographical measurements showed significantly greater improvement in the adjunctive locally delivered zoledronate sites compared to the adjunctive placebo gel with SI. When considering the radiographic measurements in healthy patients, the test group receiving adjunctive zoledronate showed a statistically greater reduction in defect metrics as well as plaque index, relative attachment level and PPD (*p* < 0.05). The difference still favored the test group receiving the adjunctive gel compared to the control in diabetic patients for radiographic measurements, relative attachment level and PPD (*p* < 0.05). No adverse events were reported. The investigators reported that the locally delivered zoledronate can be used at a low concentration such that its effect against osteoclast is maintained without affecting viability of pre-osteoblasts and fibroblasts.

Ramanauskaite et al. also followed patients in their initial phase of therapy, including repeated application of NaOCl amino acid–buffered gel prior and during SI in the test group while the control group received SI alone [33]. The treatment group also received locally applied hyaluronic acid (HA) after SI. The research group found that CAL, BOP and PPD were significantly more improved in the test group (*p* < 0.001) compared to the control, as well as the number of residual pockets (*p* < 0.001). Further analysis was performed on this sample of patients [34] to analyze microbiological changes due to treatment. The test group showed a significant favorable difference in *P. gingivalis* (*p* < 0.001), *Treponema denticola* (*p* < 0.001) and *Prevotella intermedia* (*p* < 0.001) following the use of the NaOCl gel. Wallin-Bengtsson et al. is the only research group discussed here that did not find any difference with the use of NaOCl amino acid–buffered gel (*p* > 0.05), despite application of the gel prior to SI as well as immediately after completion of SI [35].

Ariel et al. investigated the adjunctive effect of a thermosensitive formulation of a HA gel, which was provided to the test group twice; immediately following SI and one month after treatment [36]. The control group received SI alone. They saw significantly greater reduction in PPD, BOP and gain in CAL (*p* < 0.0001) with the use of the adjunctive gel. The authors also reported that for initially deep and moderate pockets, significantly more pocket closure (*p* < 0.0001) was achieved with the adjunctive gel compared to the control protocol. Pocket closure is defined as a PPD of 4 mm or less in the absence of BOP.

Rapone et al. investigated a protocol which included ozone delivery through a comprehensive full mouth disinfection protocol, including ozone at multiple steps [37]. This group found that PPD, CAL and BOP were significantly improved (*p* < 0.0001) in the test group compared to the SI alone control group. Interestingly, ozone within a gel was used by Scribante et al., with repeated application at home by the patients in the test group [38]. Patients in the control group were given a CHX gel to use at home. Both treatments, CHX gel and ozone gel, performed the same regarding all clinical parameters (*p* > 0.05). Taalab et al. used an adjunctive tea tree oil gel to SI in their test group [39]. The results favored the local delivery group, with a greater reduction in matrix metalloproteinase 8, and greater improvement in CAL after six months of follow-up (*p* < 0.05) compared to SI alone.

Omar et al. compared metronidazole delivered in periodontal pockets using patient blood–derived leukocyte platelet rich fibrin (L-PRF) to L-PRF alone [40]. CAL and PPD improved similarly between both groups (*p* > 0.05), while the modified gingival index (*p* < 0.001) were more significantly decreased in the combined PRF metronidazole group. Ilyes found that a placebo gel did as well as a gel containing doxycycline or the combination piperacillin/tazobactam, even when considering the numbers of key periodontal-associated bacterial species in addition to PPD, CAL and BOP (*p* > 0.05) [41].

A few authors investigated the effect of adjunctive local agents in residual pockets of patients enrolled in maintenance [42,43,44,45]. Benyei et al. investigated the adjunctive use of NaOCl gel compared to SI alone and found that CAL and PPD improvements were significantly greater (*p* < 0.001) in the test group [42]. Bertl et al. used a logistic regression analysis to evaluate predictors of pocket closure over twelve months of follow-up, comparing one protocol based on non-crosslinked HA gel (test) compared with a control consisting of a placebo saline solution [43]. While a clear advantage could not be seen when comparing the test to the control protocol (*p* > 0.05), the presence of plaque at twelve months had an odds ratio of 7.938 (95%CI 4.122–15.284), making it much more unlikely to obtain pocket closure in the presence of plaque (*p* < 0.001). Pilloni followed patients for twelve months in a split mouth approach, randomizing one half of the residual pockets to a test group including adjunctive polynucleotides and HA in a gel compared to SI alone in the control group [44]. They found that clinical responses were equivalent in most parameters (*p* > 0.05), except for the modified sulcus bleeding index that showed a greater improvement in the test group, which was only statistically significant in sites with PPDs of 6 mm or greater at baseline (*p* = 0.04). Radulescu et al. included a NaOCl gel as well as an adjunctive CHX gel test group [45]. The test groups in this study were compared to a control consisting of SI with a placebo gel. Microbiological tests, as well as PPD reduction and CAL gain, did not show significant differences between tests and control groups at twelve months of follow-up (*p* > 0.05). The study still reported that BOP was significantly reduced in sites treated with the NaOCl gel compared to CHX and placebo at twelve months of follow-up (*p* = 0.01). Pocket closure percentage at twelve months of follow-up was also reported to be greatest in the NaOCl test group (77.5%), compared to both the CHX group (63.16%) and the placebo control group (59.72%) (*p* = 0.044).

## 4. Discussion

### 4.1. Adjunctive Antimicrobial Photodynamic Therapy

Photodynamic therapy is based on the principle that a light source of a specific wavelength can interact with a photosensitizer to affect targeted tissues or cells [46]. The combination leads to the creation of reactive oxygen species from available molecular oxygen. These reactive oxygen molecules in turn may cause damage to cells, including bacterial cells. Immunological effects have also been described following photodynamic therapy, depending on the chosen combination and area treated. In periodontitis treatment, reactive oxygen species generated with antimicrobial photodynamic therapy (aPDT) lead to the local breakdown of bacterial cell walls in the periodontal pocket [47]. The studies included in this review based their intervention on phenothiazinium compounds (methylene blue, phenothiazine chloride), ICG and chloro aluminum phthalocyanine used as photosensitizers. The typically anaerobic periodontal pocket has been driving research toward finding alternatives to phenothiazinium class photosensitizers, with ICG being reported to still be effective when oxygen availability is reduced [20].

Interestingly, only one trial used the photosensitizer alone as an adjunctive irrigate in their control group [18]. Variation in protocols here included rinsing excess photosensitizer prior to irradiation while another study rinsed after exposing the subgingival environment to the laser irradiation [16,18]. Considering that the former found no difference between the test and the control while the latter found a significant advantage in all clinical parameters that favored the test group, the slight difference in application could potentially explain the difference in outcomes measured. Both investigators used the same diode laser and photosensitizer over multiple visits, which means that such small details in the sequence of application of aPDT may have significant effects on the clinical outcome.

With the understanding that methylene blue may present limited production of reactive oxygen species in the anaerobic periodontal pocket, Cláudio et al. tested an oxygen-releasing gel and that same gel paired with aPDT in the treatment of diabetic patients presenting residual pockets while enrolled in supportive periodontal therapy [25]. The gel was also reported on its own to release small quantities of hydrogen peroxide, which have been shown to inhibit periodontal pathogens. The level of glycemic control was an important source of variation between studies, with some authors surprisingly selecting purposefully uncontrolled diabetics with an A1c percentage greater than 7% [17,25]. Other investigators included better-managed diabetic patients [23] and patients that did not have diabetes in both test and control groups [24]. Diabetes has been described as being the cause of dysfunction in the inflammatory and immune responses of patients especially when glycemia is not well controlled [48]. In the context of periodontitis, uncontrolled diabetes is believed to lead to greater numbers of periodontal disease–associated bacteria. This is potentially accompanied by the establishment of a more destructive inflammatory milieu with increased production of pro-inflammatory cytokines and advanced glycation end products seen in diabetes. In this regard, locally delivered compounds that decrease pathogenic bacteria or the host destructive effects of the inflammatory response present an option to optimize the result of non-surgical therapy in diabetic patients.

Generally, adjunctive aPDT may present a clinical benefit in further improving PPD, CAL and BOP. This effect seems to be more consistent in the initial treatment of periodontitis as opposed to its use in residual pockets of patients undergoing supportive periodontal therapy. Most protocols supporting this potential benefit used repeated ICG with a diode laser setting between 810 nm and 909 nm [16,20,21]. Specifically in type 1 and type 2 diabetes, there does not seem to be a distinct advantage for SI with adjunctive aPDT compared to SI alone, whether it is in the initial treatment phase or during maintenance in residual pockets, since only one report showed a statistically significant difference in clinical parameters [23], with a single application of the above-mentioned ICG using an 810 nm diode laser.

### 4.2. Adjunctive Locally Delivered Agents

**Figure 2:**
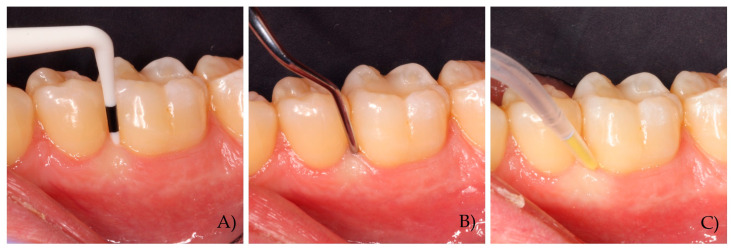
Clinical photographs depicting (**A**) a first mandibular molar presenting a 6 mm PPD (**B**) undergoing subgingival instrumentation with a mini-5 Gracey curette (11/12, Hu-Friedly) followed by (**C**) the application of local delivery of minocycline microspheres (Arestin©).

Local delivery presents the opportunity for the active compound to reach the target site, at an adequate drug concentration and for an appropriate duration of action [49]. Early dental gels typically would be expected to follow first-order kinetics within the pocket and present a rapid diminution of the concentration for the contained agent. On the other hand, controlled delivery systems allow a steady and adequate therapeutic concentration to be maintained. Vehicles also affect the ease of use as well as compound stability which affects the feasibility of this non-invasive adjunctive therapy. For example, minocycline has been used within a controlled delivery method in periodontal pockets. The antibiotic here is encapsulated in microspheres of polygly-colide-co-lactide, which hydrolyzes when in contact with water in the periodontal pocket, releasing minocycline to maintain an adequate concentration of the antibiotic in the periodontal pocket for up to two weeks [11]. Clinical photographs depicting a first mandibular molar presenting with an 8 mm PPD undergoing SI with a mini-5 Gracey curette (11/12, Hu-Friedly) followed by the application of local delivery of minocycline microspheres (Arestin©) are presented in Figure 2.

Regarding the vehicles, it is important to note that most reports did not include specific information on the fabrication and components of the local delivery products tested. Gonde et al. did report the production method of their 1% melatonin gel [29]. They reported the use of propylene glycol diluted 1:4 in distilled water, preserved with methylparaben and propylparaben, followed by sonication and addition of 1% Carbopol 934P. One day later, raw melatonin was added in solution prior to addition of triethanolamine. Another example of a well described protocol was provided by Raj et al. They used a similar process to create a 0.05% zoledronate gel using Carbopol 934P, phosphate buffer saline solution, triethanolamine (used to neutralize pH), methylparaben and propylparaben [32]. Raj et al. and Gonde et al. are great examples of moving away from the focus on antimicrobial agents to compounds with known biomodulating properties. Other protocols did not report how they fabricated the experimental product or even lacked control over the concentration of the active ingredient investigated. This was the case for the *Salvadora persica*–infused gel tested by Niazi et al. Another trial, by Bertl et al., tasked patients to repeat the application of the adjunctive agent (test or placebo) daily for three months on their interdental brush [43]. Despite such repeated application, no significant difference could be detected to support the use of this HA gel. The trial by Ariel et al., on the other hand, found a statistically significant improvement in the testing of a HA gel, and described key differences in their vehicle that could potentially explain the difference in the outcome seen [36]. Ariel et al. used a thermosensitive gel that became more viscous within the gingival pocket and included an antimicrobial preservative, octenidine. This investigation did not include the vehicle alone in its control, not allowing the distinction between the effect of the HA and the antimicrobial preservative within the carrier.

Plant derived compounds were used by three authors [21,31,39]. These trials reported clinical improvements that were still statistically significant after at least six months of follow-up, showing the potential benefit of adjunctive locally delivered *Salvadora persica*, tea tree oil and *Aloe vera* to SI. Many other potential agents exist that could be used for their biological activity as well as their antimicrobial effects to improve the healing during and after periodontitis treatment. A recent review gathering evidence on the use of natural products in all spheres of dental sciences and therapies showed that investigations have focused on studying the response of patients to at home oral rinses based on natural products, which has included randomized controlled trials for the treatment of gingivitis [50]. More investigations on the effects of plant-derived products in mouth rinses in the setting of periodontitis treatment would be valuable and could potentially lead to the discovery of new therapeutic compounds.

Iorio-Siciliano et al. investigated an amino acid–buffered NaOCl gel [30]. The gel was applied for 30 s in pockets of at least 5 mm depth, followed by MINST, a technique of SI based on magnification and micro instrumentation to optimize wound healing. Patients were thereafter recalled monthly for six months for cleaning and oral hygiene reinforcement. This close recall schedule may influence the overall result of the study and is yet another difference that limits the comparisons that can be drawn from other trials on this same NaOCl gel [33,42,45].

The NaOCl gel was reported to help soften calculus to facilitate instrumentation while also decontaminating the root surface [30]. Amino acids glutamic acid, leucine and lysine are also included in the gel to promote survival and attachment of periodontal ligament cells on root surfaces after instrumentation [30]. Another source of variability between studies stems from the fact that some investigators included the studied agent as part of a comprehensive disinfection protocol including multiple steps [30,37]. Iorio-Siciliano’s research group studying adjunctive effects to MINST included a very strict post-treatment professional plaque control regimen, which included two weeks of CHX rinsing in addition to the previously mentioned supragingival professional plaque removal visits [30]. Rapone et al. used an ozone-based disinfection protocol, which includes ozone in rinses and irrigation during the initial phase of therapy [37].

We can also note that researchers seldom include patient-centered criteria to evaluate the success of therapy. Patient-centered criteria are important considerations since the potential advantage of local delivery at the time of non-surgical treatment is to limit the need of more invasive treatment associated with morbidity. An exception to this was Wallin-Bengtsson et al., a report that included oral health–related quality-of-life questionnaires in their study design and followed patients up to twelve months [35].

### 4.3. Study Limitations

This narrative review included reports from randomized controlled trials published in the last five years retrievable from PubMed using a customized search query. Articles focusing on the local use of pharmaceutical agents in the context of periodontal surgery and dental implant therapy were excluded, which limits the scope of the discussion to non-surgical treatment of periodontitis. Due to highly variable clinical protocols, direct comparison of clinical results obtained between studies is limited. Blinding of the participants and investigators in the studies was variable, with most authors reporting that blinding of patients when using a local delivery vehicle or aPDT is not feasible due to the additional manipulation required by the application of a gel or laser. One study found a solution to blind patients to group allocation by establishing a sterile field and draping the subjects prior to SI [33]. Most trials failed to include a placebo in their control arm, which in most cases would have been a formulation of the used hydrogel without the active compound investigated. When it came to aPDT, most studies did not include a placebo in their control or a group receiving photosensitizer irrigation without laser irradiation. Some investigators also chose to include a positive control only, using adjunctive agents such as locally delivered CHX gel, systemic antibiotics or even a surgical intervention facilitating the SI. Such protocols would be more easily comparable to other studies if they included a negative control consisting of SI with the vehicle alone as placebo. Trials following a greater number of patients with protocols describing any adverse events encountered are needed. This would allow an appreciation of the unintended impact of compounds and protocols on patients such as potential allergies and gastrointestinal disturbances. Some consequences may affect the population at large, for example, through the emergence of microorganism resistance.

### 4.4. Future Perspectives

Periodontal treatment methods are being developed to achieve sustained delivery and maximize the effectiveness of known compounds at targeted treated sites. An impressive variety of nanoparticles can be used as part of delivery pharmacological compounds. These include chitosan, alginate, xanthan gum, cellulose, polymeric micelles, dendrimers, inorganic particles, nanocrystals, metallic nanoparticles, quantum nanoparticles, quantum dots, organic nanoparticles (proteins and polysaccharides) and liposomes [51]. Future innovation could arise from the use of liposomes in the development of vehicles for local delivery of antimicrobial agents. CHX and triclosan have been successfully delivered in vesicles since the 1990s, with Jones, for example, showing that this method led to a more effective killing of *Streptococcus epidermidis* [52]. This method of encapsulation within amphiphilic molecules can help reduce irritation caused by active agents and act as a source for sustained release of the contained medication. Vesicular delivery through liposomes in periodontitis is in its early development, with theoretical proof of concept that liposomes may be able to even reach deep within dentin tubules that are contaminated in periodontitis [53]. The use of liposomes allows delivery of pharmaceutical agents presenting poor water solubility. Future research efforts could investigate further the adaptation of liposomes in overall delivery of medications, as well as improved predictability and simplicity in the fabrication of vehicles relying on liposomes [54]. With the great variety of emerging nanoparticle systems for drug delivery, advances in this field could allow more effective use of locally delivered agents used in the past for periodontitis, such as CHX, doxycycline and minocycline, and the investigation of new compounds normally poorly soluble.

## 5. Conclusions

The current narrative review has focused on the latest progress in locally delivered therapy in the non-surgical treatment of periodontitis. Much of the emphasis in the last five years has been on locally delivered antimicrobials like a NaOCl gel buffered with amino acids (Perisolv©), which was found to provide a statistically significant clinical benefit in most clinical trials. In this review, multiple techniques utilizing diode lasers and locally deposited photosensitizers were reported, precluding the identification of one single protocol being superior to the others. Positive results on diode lasers combined with ICG and, to a lesser extent, phenothiazine chloride photosensitizers, warrant further research. Trials also demonstrated the use of improved vehicles to optimize results of previously available products, such as HA in a thermosensitive gel. Future research is needed to establish an optimal protocol to predictably improve non-surgical outcomes for patients affected by periodontitis using aPDT. Investigators should also research different vehicles, which may allow present, and future, locally delivered agents to show greater clinical effectiveness, as well as a more sustained release in the subgingival environment for the non-surgical treatment of periodontitis. Future research should focus on including relevant controls such as adjunctive placebos. These should include the chosen vehicle, or the photosensitizer alone deposited in the periodontal pockets. This is important since a photosensitizer presents antimicrobial effects of their own, which is part of what allows their localization to bacteria before laser activation, with no reported emergence of resistance to the resulting reactive oxygen species [47]. On the other hand, antibiotics tend to be more specific in their bactericidal or bacteriostatic effect, which can allow the emergence of resistant pathogens. This is believed to be greatly reduced in locally delivered antibiotics [10]. In conclusion, a variety of adjunctive aPDT protocols and locally delivered agents present statistically significant benefit compared to SI alone when it comes to clinical parameters assessed in the response to periodontitis treatment. More evidence is necessary to evaluate if these differences are clinically significant and cost-effective.

## Figures and Tables

**Table 1:** Methods and results of clinical trials on adjunctive antimicrobial photodynamic therapy with subgingival instrumentation (*n* = 13).

Author	*n*	Randomization	Population	Test Group	Control/Placebo	Results
Al-Khureif [15]	17	Patients	Initial treatment, Periodontitis Stage III–IV Grade C	SI + [670 nm DL + PTC aPDT on days 1, 3, 7 and 14]	SI + AMOX/MET (500 mg/500 mg) tid for 7 days.	At sites with baseline PPDs of 7 mm or greater, significantly greater reduction in PPD (*p* = 0.0013) and CAL gain (*p* < 0.001) at six-month follow-up favored the test group. GCF cytokines changes (reduced IL-17, increased IL-10 both *p* < 0.05) favored the control group.
Sukumar [16]	30	Split mouth mandibular posterior sextants	Initial treatment, Periodontitis Stage II Grade A	SI + [810 nm DL + ICG aPDT days 7, 14, 28]	SI alone	PI (*p* < 0.001), GI (*p* < 0.001), BOP (*p* < 0.05), PPD (*p* < 0.001) and CAL (*p* < 0.05) presented statistically significant improvements in favor of the aPDT group compared to SI alone. There was also a significantly greater reduction in periodontal pathogens (*p* < 0.05) at six months in the aPDT group.
Cláudio [17]	31	Patients	Initial treatment T2D A1c > 7%, Periodontitis Stage III–IV Grade C	SI + [660 nm DL + MB aPDT on days 1, 2 and 4]	SI alone	No statistically significant difference (*p* > 0.05) between the improvement in PPD, CAL and BOP when comparing the test and control group. Levels of *Pg* and *Pi* were unchanged in both groups.
Annunziata [18]	24	Split mouth, periodontal pockets	Initial treatment, Periodontitis Stage II–III	SI + [810 nm DL + ICG aPDT on days 7 and 28]	SI + ICG irrigation	No statistically significant difference between improvement in PPD, CAL, and BOP after six months of follow-up when comparing the test and control group (*p* > 0.05).
Andere [19]	46	Patient	Maintenance, Periodontitis Stage III–IV Grade C	SI + [660 nm DL + MB aPDT on days 1, 2, 7 and 14]	OFD	OFD resulted in greater PPD reduction (*p* = 0.001), GR (*p* = 0.001), early dentin hypersensitivity (*p* = 0.03) and postoperative pain (*p* = 0.03) compared to aPDT. CAL gain was similar in both test and control groups (*p* > 0.05).
Costa [20]	24	Split mouth, randomized quadrants	Maintenance, Periodontitis Stage II–III, Grade A	SI + [909 nm DL + ICG aPDT on days 1 and 15]	SI + Sham aPDT/saline irrigation	Statistically significant difference of improvement in BOP (*p* = 0.046) and PISA (*p* = 0.001) after six months of follow-up supported the use of the test treatment when compared to the control. CAL and PPD improvement were similar in both groups (*p* > 0.05).
Niazi [21]	73	Patients	Initial treatment, Periodontitis Stage II–III	SI + [*Sp* gel] or [810 nm DL + ICG aPDT]	SI alone	In initially deep sites, aPDT group provided significantly greater CAL and PPD improvement measured at six months of follow-up compared to the control and *Sp* groups (*p* < 0.05). The *Sp* gel group presented a statistically significantly greater reduction in BOP at six months (*p* < 0.05) compared to aPDT and the control group.
Katsikanis [22]	21	Split mouth, randomized quadrants	Initial treatment, Periodontitis Stage III Grade A–B	SI + [670 nm DL + MB aPDT] or [940 nm DL]	SI alone	No statistically significant difference in CAL, PPD and BOP improvements between test and control groups at six months of follow-up (*p* > 0.05).
Al-Momani [23]	50	Split mouth	Initial treatment T2D, Periodontitis Stage III–IV Grade B–C	TG1: A1c < 6% TG2: T2D, A1c 6–10% TG3: T2D, A1c > 10% SI + 810 nm DL + ICG	CG1: A1c < 6% CG2: T2D, A1c 6–10% CG3: T2D, A1c > 10% SI alone	TG1 vs CG1: CAL gain and PPD reduction greater in aPDT group (*p* < 0.05). TG2 vs CG2: CAL gain, PPD and BOP improvement greater in aPDT group (*p* < 0.05). TG3 vs CG3: CAL and BOP improvement greater in aPDT group (*p* < 0.05), PPD reduction was similar in both groups (*p* > 0.05).
Cunha [24]	38	Patients	Initial treatment, T1D, Periodontitis Stage III–IV Grade A	TG1: DSRP TG2: DPDT SI + [650 nm DL + MB aPDT on days 1, 7, 14]	CG1: CSRP CG2: CPDT	No statistically significant difference in improvements measured in CAL, PPD and BOP at six months for SI with compared to SI without adjunctive aPDT (*p* > 0.05), with overall healthy control patients improving more than T1D patients in the test group regarding PPD reduction after aPDT treatment (*p* < 0.05).
Cláudio [25]	45	Patients	Maintenance T2D A1c > 7% Periodontitis Stage III–IV Grade C	SI + [oxygen releasing gel + 660 nm DL + MB] or [oxygen releasing gel]	SI alone	Improvement in PPD, CAL, BOP did not support the use of the test intervention at six-month follow-up in this group of poorly controlled diabetic patients (no statistically significant difference between test and control groups with *p* > 0.05).
De Araújo [26]	63	Residual pockets	Maintenance, Periodontitis Stage III	SI + [660 nm DL + CAPC aPDT]	SI + saline irrigation	No statistically significant difference in PPD, CAL and BOP after six months of follow-up in treated residual pockets (*p* > 0.05). Test pockets did see a statistically significant difference compared to control sites, with a greater improvement of gingival bleeding index (*p* = 0.041).
Schär [27]	40	Patients	Maintenance, Periodontitis Stage II–III	SI + [760 nm DL + PTC tg-aPDT]	SI alone	Similar improvements in both groups were seen at six months of follow-up for CAL and PPD (no statistically significant difference between test and control with *p* > 0.05). The aPDT test group showed a statistically significant greater reduction in BOP compared to the control group (*p* < 0.05).

ICG: indocyanine green. PTC: phenothiazine chloride. MB: methylene blue, CAPC: chloro aluminum phthalocyanine, DL: diode laser, AMOX: amoxicillin, MET: metronidazole SI: subgingival instrumentation, PPD: periodontal pocket depth, CAL: clinical attachment level, BOP: bleeding on probing, GI: gingival index, PI: plaque index, PISA: periodontal inflamed surface area, aPDT: antimicrobial photodynamic therapy, tg: transgingival, Sp: *Salvadora persica*, GCF: gingival crevicular fluid, TG: Test group, CG: control group, T1D: type 1 diabetes, T2D: type 2 diabetes, A1c: glycated hemoglobin percentage, OPD: open flap debridement, DSRP: Diabetic patient receiving scaling and root planing, DPDT: Diabetic patient receiving scaling and root planing with aPDT, CSRP: Normoglycemic patient receiving scaling and root planing, CPDT: Normoglycemic patient receiving scaling and root planing with aPDT, GR: gingival recession.

**Table 2:** Methods and results of clinical trials on adjunctive locally delivered agents with subgingival instrumentation (*n* = 18).

Author	*n*	t	Randomization	Population	Test Group	Control/Placebo	Results
Barahim [28]	24	6 mo	Patients	Initial treatment T2D A1c < 7% Periodontitis Stage III Grade B	SI + OZ gel	SI alone	Statistically significant greater reduction in VAS depiction of pain at seven days after SI (*p* = 0.017) and of PDL ligament width on periapical radiograph (*p* = 0.014) at the six-month follow-up compared to the control group.
Gonde [29]	22	6 mo	Split mouth, intrabony defect pair	Periodontitis, intrabony defects Periodontitis Stage III	SI + Melatonin gel	SI alone	Statistically significant differences favoring the test group were observed in bone fill visible on CBCT, as well as CAL and PPD improvement compared to the control group (*p* < 0.05).
Iorio-Siciliano [30]	40	6 mo	Patients	Initial treatment, Periodontitis Stage III–IV Grade A–B	MINST + NaOCl gel	MINST alone	Test group presented statistically significant increase in percentage of pocket closure and residual pockets without bleeding compared to MINST alone (*p* = 0.001). This was also the case for PPD reduction and CAL gain (*p* = 0.001)
Qamar [31]	150	6 mo	Patients	Initial treatment, Periodontitis Stage II–III	SI + [810 nm DL + ICG aPDT] or *Aloe vera* gel	SI alone	Statistically significant improvement in cytokine profiles (*p* < 0.05) maintained at six months in both treatment groups compared to the control. PI, BOP, PPD and CAL improvements favored the *Aloe vera* gel group when compared to both aPDT and SI alone (*p* < 0.05). BOP reduction was still greater in the aPDT group compared to control (*p* < 0.05). In deep pockets (>6 mm), the aPDT test group presented a greater improvement in CAL compared to both control and *Aloe vera* gel groups (*p* < 0.05).
Raj [32]	60	6 mo	Randomized intrabony pockets	Initial treatment, Periodontitis Stage III Grade B	G1: Periodontitis alone, G2: Periodontitis and controlled T2D (A1c < 7%). SI + zoledronate gel	SI + placebo gel	At six months, PI, PPD, RAL, DD on IOPA and CBCT as well as DDR% improved more in test compared to placebo in non-diabetics (statistically significant with *p* < 0.05). In diabetics, this was still true for PPD, RAL, DD on IOPA and CBCT as well as DDR% (*p* < 0.05).
Ramanauskaite [33]	48	6 mo	Patients	Initial treatment, Periodontitis Stage II–III Grade A-B	Pre SI NaOCl, repeated 2–3 times + CL-HA gel in sterile field	SI alone with sterile field	CAL, BOP and PPD reduction favored the test (*p* < 0.001), but test group also had statistically significant better hygiene at end of follow-up (*p* < 0.001). The percentage of residual pockets 4–6 mm (*p* < 0.001) and residual 7 mm+ favored the test as well (*p* = 0.003).
Ramanauskaite [34]	48	6 mo	Patients	Initial treatment, Periodontitis Stage II–III Grade A–B	Pre SI NaOCl, repeated 2–3 times + CL-HA gel in sterile field.	SI alone with sterile field	At six months, less frequent detection of Pg (*p* = 0.034), Td (*p* < 0.01) and Pi (*p* = 0.02) was found in the test compared to the control, the latter did not show a significant improvement.
Wallin-Bengtsson [35]	38	12 mo	Patients	Initial treatment, Periodontitis Stage I–IV, Grade A–C	SI + Pre and post SI NaOCl	SI alone	No difference in clinical parameters or mean OHIP scores between the test group and control (*p* > 0.05).
Ariel [36]	34	6 mo	Split-mouth design	Initial treatment, Periodontitis Stage III	SI + thermosensitive gel with an active HA, repeated at 1 month in PPD 5 mm or more	SI alone	Statistically significant greater improvement in BOP, CAL and PPD in the test group compared to SI alone at six months (*p* < 0.0001).
Rapone [37]	90	6 mo	Patients	Initial treatment Periodontitis Stage II–IV, Grade A–B	SI + OZ irrigation protocol	SI alone	Statistically significant improvements in PPD, CAL and BOP favored the test compared to SI alone (*p* < 0.0001).
Scribante [38]		6 mo	Split mouth design	Initial treatment, Periodontitis Stage III	SI + OZ gel repeated at home.	SI + CHX gel repeated at home	Ozone gel performed as well as CHX gel in CAL, PPD, BOP, PCR. Rec and TM were maintained regardless of type of treatment (*p* > 0.05).
Taalab [39]	30	6 mo	Patients	Initial treatment Periodontitis Stage II Grade B	SI + TTO	SI alone	Statistically significant improvement favored the TTO gel when measuring MMP-8 levels, BOP and CAL (*p* < 0.05).
Omar [40]	24	6 mo	Patients	Initial treatment, Periodontitis Stage II	SI + L-PRF + METRO	SI + L-PRF	Modified gingival index decrease also favored the test group (*p* < 0.001). CAL, PPD and BOP improved similarly between the test and control group after six months (*p* > 0.05).
Ilyes [41]	64	6 mo	Patients	Initial treatment, Periodontitis Stage III–IV	SI + PTG or DOX	SI + placebo gel	PPD, CAL and BOP showed no significant difference between the test and the control group (*p* > 0.05). Same conclusions were drawn from quantification of periodontal pathogen following treatment.
Benyei [42]	50	9 mo	Patients	Maintenance, Periodontitis Stage III–IV	SI+ adjunctive NaOCl gel at residual pockets	SI alone	Greater improvement in CAL and PPD observed in the test group at nine months of follow-up (*p* < 0.001). BOP presented similar improvements in both test and control groups (*p* > 0.05).
Bertl [43]	56	12 mo	Pockets	Maintenance, Periodontitis Stage III–IV Grade B–C	HA gel repeated daily supragingival for 3 months.	SI + placebo saline	Multivariable regression analysis showed that presence of plaque was a significant predictor of persistent disease at a site (7.938 OR with *p* = 0.001). Regarding clinical parameters, both treatments performed the same (*p* > 0.05).
Pilloni [44]	50	12 mo	Split mouth, randomized residual pockets	Maintenance, Periodontitis Stage III	SI + Polynucleotides and HA	SI alone	Significantly greater reduction in mSBI in sites at baseline PPD 6 mm or more after one year of follow-up (*p* = 0.004), otherwise no statistically significant difference was seen between test and control groups (*p* > 0.05).
Radulescu [45]	62	12 mo	Patients	Maintenance, Periodontitis Stage III–IV	SI + NaOCl gel or CHX gel	SI + placebo gel	Pocket closure percentage showed a significant advantage for the test compared to the control group and CHX gel group up to twelve months post SI (*p* = 0.044). Periodontal pathogen counts reduction, GR, PPD reduction and CAL gain were similar in all three groups (*p* > 0.05).

ICG: indocyanine green, DL: diode laser, AMOX: amoxicillin, MET: metronidazole, NaOCl: sodium hypochlorite, CHX: chlorhexidine, OZ: ozone, PTG: tazobactam, DOX: doxycycline, TTO: tea tree oil, HA: hyaluronic acid, CL: cross-linked, L-PRF: leukocyte-platelet rich fibrin, Pg: *Porphyromonas gingivalis*, Pi: *Prevotella intermedia*, Td: *Treponema denticola*, PCR: plaque control record, PDL: periodontal ligament, RAL: relative attachment level, TM: tooth mobility, VAS: visual analog scale SI: subgingival instrumentation, MINST: minimally invasive non-surgical therapy, CBCT: cone beam computed topography, IOPA: intraoral periapical radiograph, DDR%: defect depth reduction percentage, DD: defect depth, PPD: periodontal pocket depth, CAL: clinical attachment level, BOP: bleeding on probing, GI: gingival index, PI: plaque index, PISA: periodontal inflamed surface area, aPDT: antimicrobial photodynamic therapy, tg: transgingival, T1D: type 1 diabetes, T2D: type 2 diabetes, A1c: glycated hemoglobin percentage, MMP: matrix metalloproteinase, GR: gingival recession.

## Data Availability

The PubMed search strategy is included within the article.

## Abbreviations

The following abbreviations are used in this manuscript:

SI	Subgingival instrumentation
PPD	Periodontal pocket depth
BOP	Bleeding on probing
CHX	Chlorhexidine
CAL	Clinical attachment level
aPDT	Antimicrobial photodynamic therapy
ICG	Indocyanine green
OFD	Open flap debridement
A1c	Glycated hemoglobin percentage
NaOCl	Sodium hypochloride
VAS	Visual analog scale
CBCT	Cone beam computed topography
MINST	Minimally invasive non-surgical therapy
HA	Hyaluronic acid
L-PRF	Leukocyte-platelet rich fibrin
